# A rare case of metastatic Merkel cell carcinoma presenting without a primary cutaneous lesion: A case report

**DOI:** 10.1097/MD.0000000000050345

**Published:** 2026-09-04

**Authors:** Na Luo, Wen Xiao, Lijun Su, Ping Chen

**Affiliations:** a Department of Oncology, General Hospital of Ningxia Medical University, Yinchuan, Ningxia, China.

**Keywords:** CK20, immunotherapy, Merkel cell carcinoma, Merkel cell polyomavirus, neuroendocrine carcinoma, occult primary

## Abstract

**Rationale:**

Merkel cell carcinoma (MCC) is a rare, aggressive cutaneous neuroendocrine malignancy. While typically associated with a primary skin lesion, cases of occult-primary MCC are uncommon and pose significant diagnostic challenges, often leading to delays in appropriate management.

**Patient concerns:**

A 44-year-old male presented with a painless, enlarging mass in the right groin of 1 month’s duration. He denied any history of preceding or concomitant skin nodules, lesions, or rashes.

**Diagnoses:**

Pathological examination of the right inguinal mass, via initial biopsy and subsequent wide excision, demonstrated a metastatic neuroendocrine carcinoma. Comprehensive immunohistochemical profiling (CK20+, CKpan+, Syn+, cluster of differentiation 56+, thyroid transcription factor 1−, with positive Merkel cell polyomavirus staining) confirmed the diagnosis of metastatic Merkel cell carcinoma. Staging positron emission tomography/CT and subsequent imaging identified widespread metastatic disease to the pancreas, abdomen, and mediastinum without evidence of a primary cutaneous lesion.

**Interventions:**

The patient underwent surgical excision of the symptomatic right inguinal nodal mass. Upon confirmation of widespread systemic progression, he received first-line systemic therapy with the etoposide + platinum regimen (etoposide + cisplatin) combined with the PD-1 inhibitor sintilimab, followed by second-line therapy with the folinic acid + fluorouracil + oxaliplatin regimen (oxaliplatin + fluorouracil) combined with sintilimab.

**Outcomes:**

The disease exhibited a highly aggressive course. Transient symptomatic relief was observed after the first cycle of first-line therapy. However, the disease progressed rapidly through both first- and second-line chemo-immunotherapy regimens. The patient died approximately 14 months after initial presentation.

**Lessons:**

This case underscores that MCC can present as metastatic carcinoma without an identifiable primary cutaneous lesion. This observation necessitates its inclusion in the differential diagnosis of metastatic neuroendocrine tumors, even in the absence of skin findings. Comprehensive immunohistochemistry, including cytokeratin 20 and Merkel cell polyomavirus status, is crucial for diagnosis. The rapid progression and poor response to combined chemo-immunotherapy in this young patient highlight the heterogeneous and often refractory nature of advanced MCC.

## 1. Introduction

Merkel cell carcinoma (MCC) is a rare and highly aggressive primary neuroendocrine carcinoma of the skin, first described by Toker in 1972.^[1]^ Its incidence is highest among the elderly and immunocompromised populations.^[2]^ Pathogenetically, both ultraviolet radiation-induced mutagenesis and clonal integration of the Merkel cell polyomavirus (MCPyV) are established etiological factors.^[3]^ The classic clinical presentation is a rapidly growing, painless, violaceous or red nodule on sun-exposed skin.^[4]^ MCC is notorious for its high rates of local recurrence, nodal involvement, and distant metastasis.^[5]^ Diagnosis hinges on histopathological findings of dermal infiltrates of small blue round cells with characteristic immunohistochemical staining, most notably for cytokeratin 20 (CK20) in a paranuclear dot-like pattern.^[6]^ Due to its rarity, variable clinical presentation, and lack of characteristic symptoms, MCC may be misdiagnosed as other cutaneous malignancies such as squamous cell carcinoma, basal cell carcinoma, melanoma, and primary cutaneous B-cell lymphoma.^[7]^ Cases without an identifiable primary skin lesion, termed occult-primary MCC, represent a diagnostic dilemma and are estimated to account for a minority of presentations.^[8]^ This case report details the clinical journey, pathological confirmation, and therapeutic management of a young patient with widely metastatic MCC arising from an occult primary, illustrating the challenges in diagnosis and the limitations of current therapeutic strategies for advanced disease.

## 2. Case report

### 2.1. Initial presentation, imaging, and preliminary biopsy

**Figure 1.**
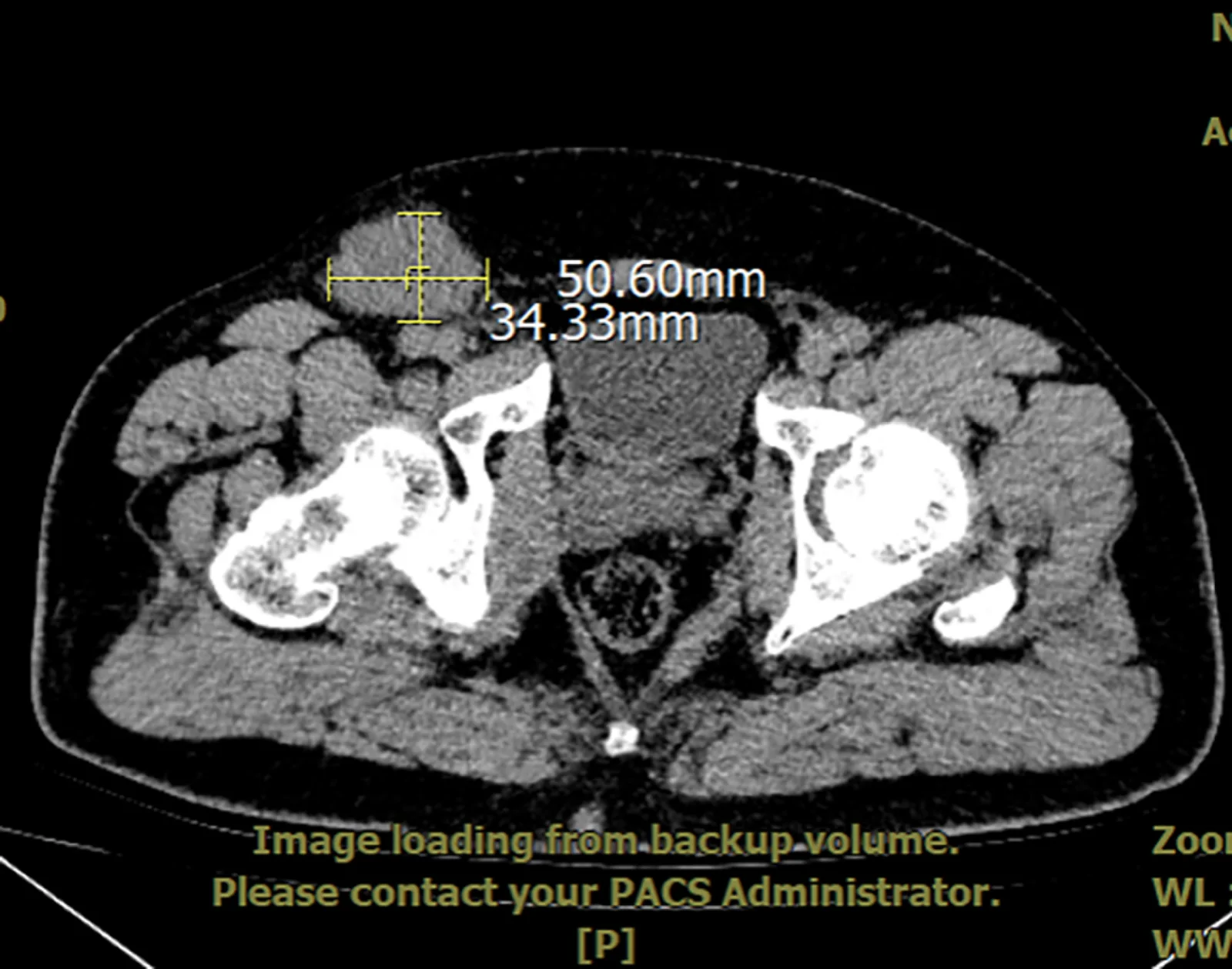
Non-contrast CT scan of the lower abdomen (February 2024) demonstrating a 5.0 cm × 3.4 cm soft tissue mass in the right inguinal region. CT = computed tomography.

A 44-year-old male presented in January 2024 with a newly discovered, painless mass in his right groin. An initial local B-ultrasound described a 4.7 cm × 3 cm solid subcutaneous mass with irregular borders and heterogeneous echogenicity. He had a 5-year history of hypertension, with a maximum blood pressure of 180/115 mm Hg, controlled with oral irbesartan hydrochlorothiazide tablets to around 140/90 mm Hg, and denied any personal or family history of malignancy or systemic skin lesions. Physical examination at our hospital in February 2024 revealed enlarged lymph nodes in the right inguinal region, measuring approximately 5 cm × 4 cm. The nodes were firm, matted together, fixed, and non-tender. A non-contrast CT scan of the lower abdomen performed at our institution confirmed a soft tissue mass measuring 5.0 cm × 3.4 cm in the right inguinal region (Fig. 1).

**Figure 2.**
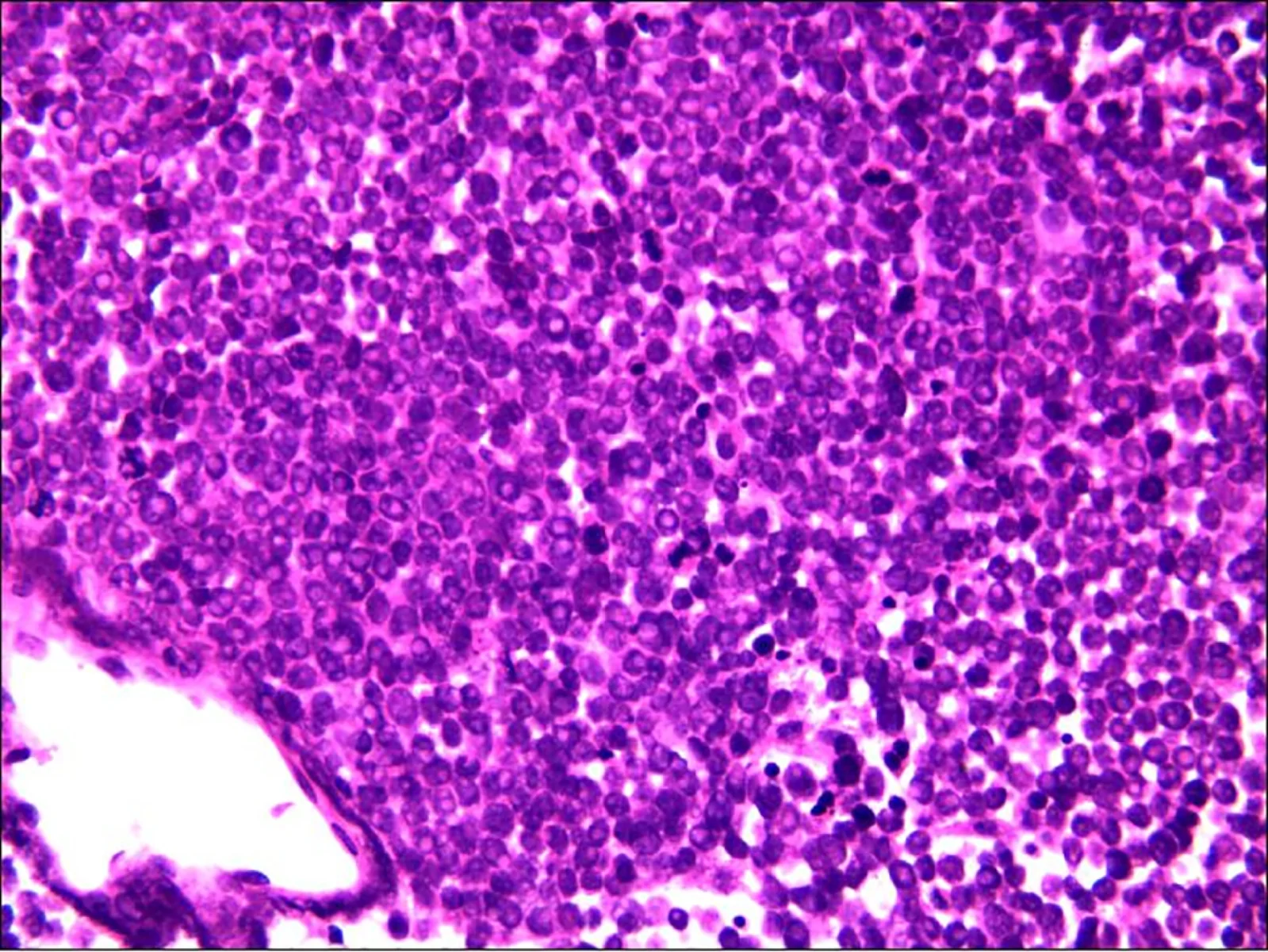
Histopathological examination of the right inguinal lymph node biopsy demonstrated diffuse infiltration by malignant small round cells within nodal tissue, suggestive of neuroendocrine carcinoma (H&E stain, ×400). H&E = hematoxylin and eosin.

On February 9, 2024, an ultrasound-guided core needle biopsy of the right inguinal mass was performed. Microscopic evaluation of the biopsy specimen showed lymphoid tissue diffusely effaced by tumor cells. Combined with immunohistochemical (IHC) staining, the findings were consistent with a neuroendocrine tumor, with small cell carcinoma favored, and metastasis could not be ruled out. The IHC results were as follows: CKpan(+), Syn(+), cluster of differentiation 56(+), Ki67 (40%+), TTF-1(−), with other markers including CD3, CD20, chromogranin A, and S100 being negative or only individually positive (Fig. 2).

### 2.2. Comprehensive staging and pathological confirmation

**Figure 3.**
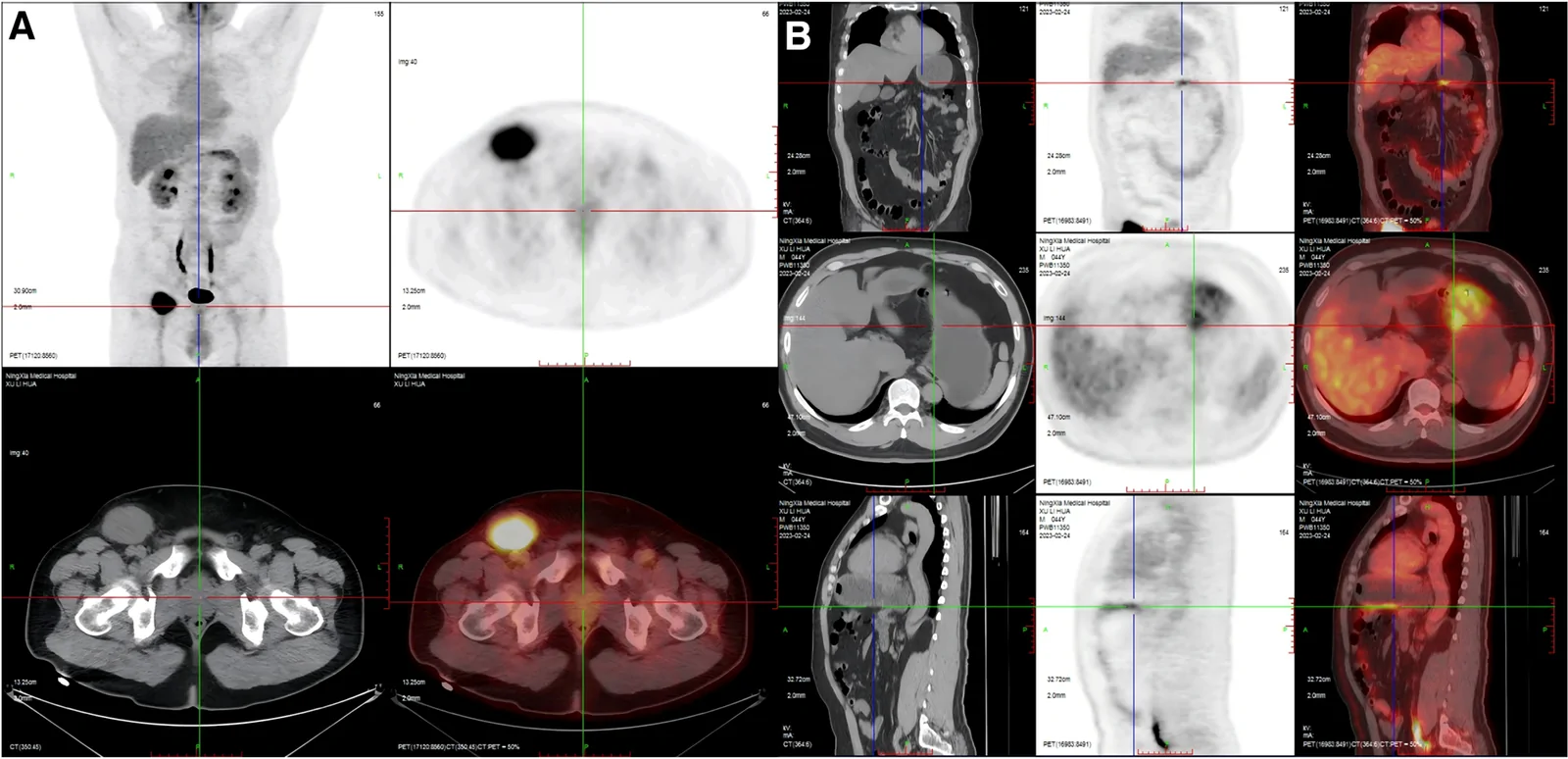
18F-FDG PET/CT scan performed on February 23, 2024. (A) Intense FDG uptake in the right inguinal lymph node, measuring 5.5 cm in maximum diameter with SUVmax 9.8. (B) Focal mild metabolic activity along the lesser curvature of the gastric body (SUVmax 4.1); subsequent gastrointestinal endoscopy revealed no malignant lesions. No other hypermetabolic foci suggestive of malignancy were identified. CT = computed tomography, FDG = fluorodeoxyglucose, PET = positron emission tomography.

To identify a primary lesion, a whole-body positron emission tomography/CT scan was performed on February 23, 2024. It showed intense hypermetabolism (SUVmax 9.8) in the enlarged right inguinal lymph nodes, consistent with a metastatic deposit (Fig. 3A). No other hypermetabolic foci suggestive of a primary skin or visceral malignancy were identified. Mild focal fluorodeoxyglucose uptake was noted in the gastric body (SUVmax 4.1), prompting a subsequent digestive endoscopy on March 6, which revealed only chronic gastritis and reflux esophagitis. Enhanced CT scans of the chest and abdomen showed only scattered sub-centimeter lung nodules and small abdominal aortic lymph nodes, none of which were characteristic of a primary tumor (Fig. 3B). Serum tumor markers (CEA, CA199) were within normal limits.

**Figure 4.**
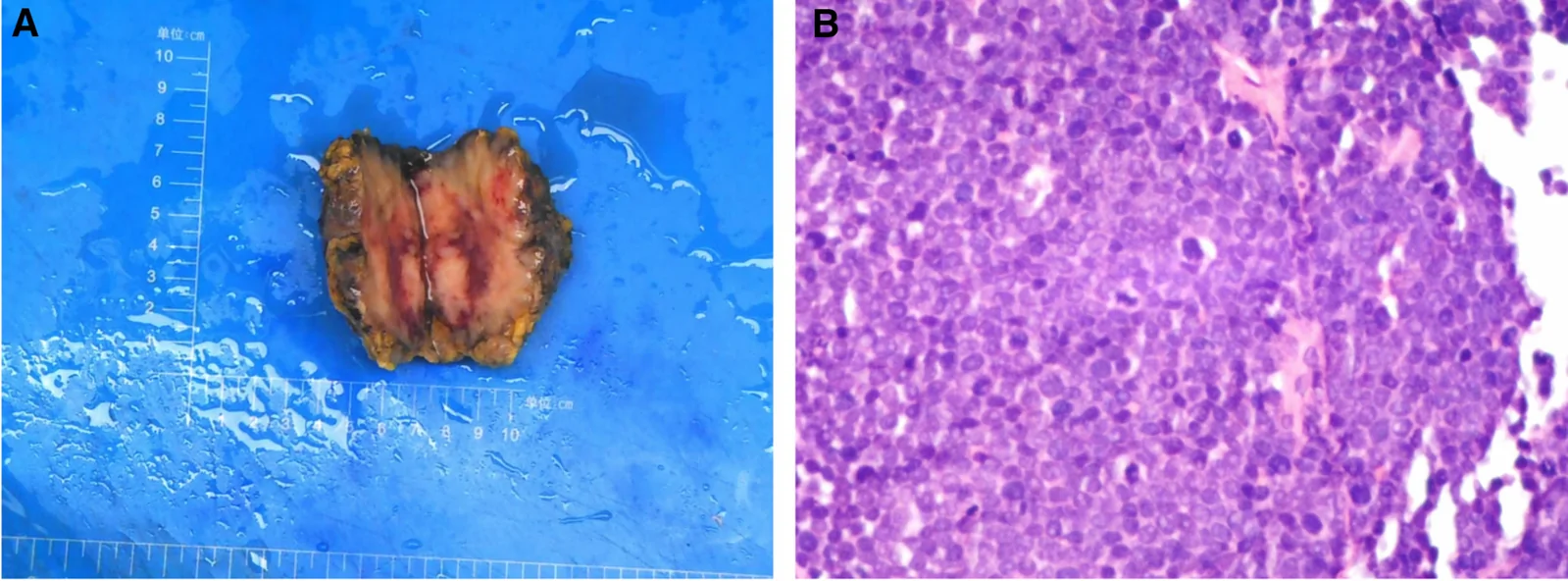
Pathological findings of the resected right inguinal mass. (A) Gross appearance of the surgical specimen, measuring 6 cm × 4 cm × 3 cm, with a grayish-white, solid cut surface. (B) Microscopic examination demonstrating diffuse infiltration of the dermal fibroadipose tissue by small blue round malignant cells with scant cytoplasm and frequent mitotic figures. Small blood vessel proliferation is observed in the stroma (H&E stain, ×400). H&E = hematoxylin and eosin.

On March 23, 2024, the patient underwent surgical excision of the right inguinal mass. Histopathological review was performed by a consultant pathologist. Microscopic examination of the excised specimen showed diffuse infiltration of dermal and subcutaneous fibroadipose tissue by sheets of small, blue, round malignant cells with scant cytoplasm, a high nuclear-to-cytoplasmic ratio, and frequent mitoses (Fig. 4). A comprehensive IHC panel was performed. The tumor cells were positive for CK20 (with a characteristic dot-like pattern), AE1/AE3, synaptophysin, cluster of differentiation 56, INSM1, and SSTR2. They were negative for thyroid transcription factor 1 (TTF-1), CDX-2, P63, and other markers. The Ki67 proliferation index was 40%. The pathological diagnosis was metastatic neuroendocrine carcinoma (NEC G3), with morphology and IHC profile highly suggestive of MCC. Postoperatively, the patient received no immediate adjuvant therapy due to a lack of symptoms.

### 2.3. Disease progression and therapeutic management

**Figure 5.**
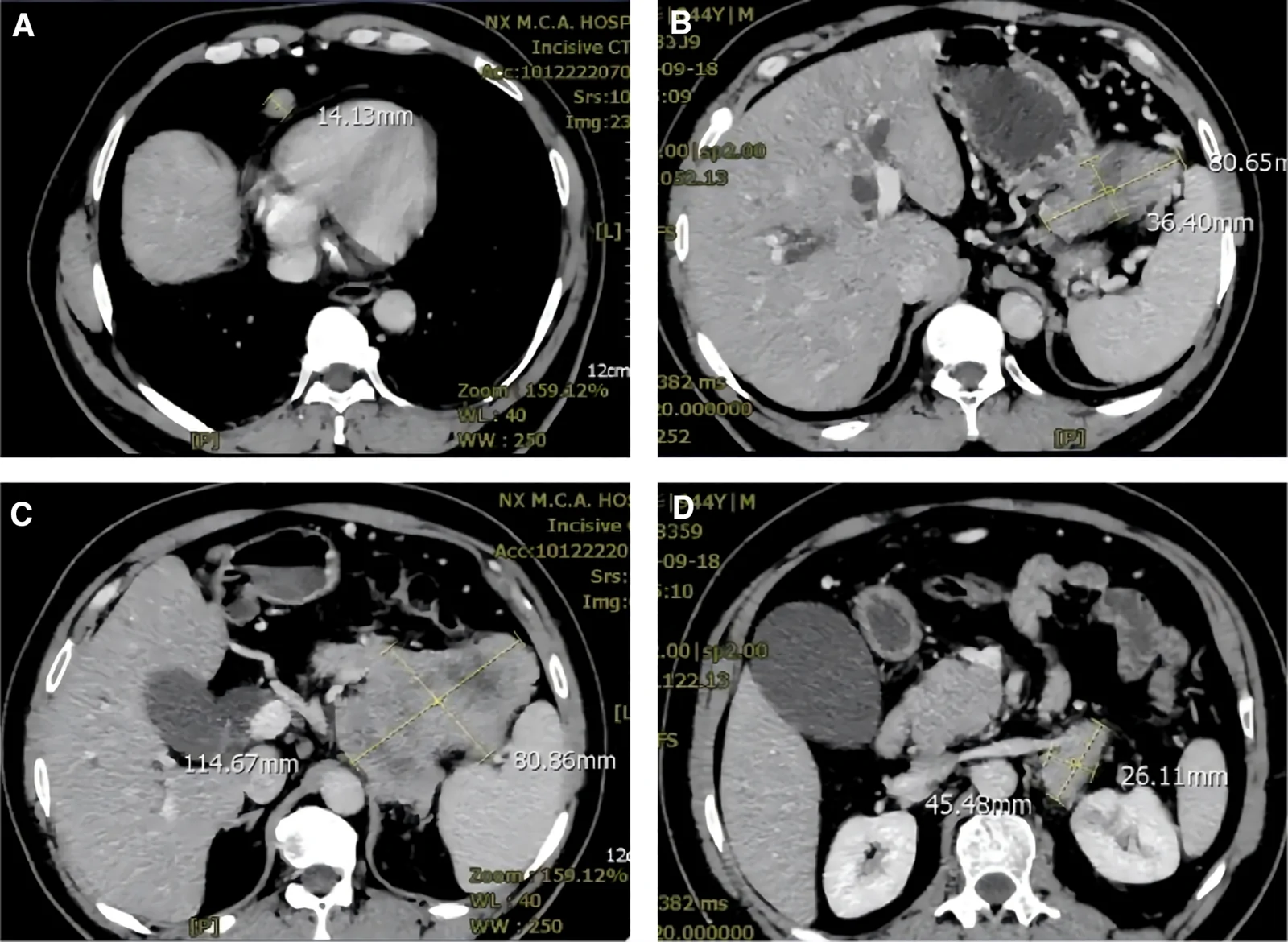
Contrast-enhanced CT of the chest and abdomen (September 2024). (A) Mediastinal lymphadenopathy. (B) Pancreatic mass. (C, D) Multiple large intra-abdominal masses. CT = computed tomography.

The patient’s clinical course is summarized in Table 1. Approximately 6 months later, in September 2024, the patient developed progressive abdominal pain, jaundice, and a 5 kg weight loss. Repeat CT imaging of the chest and abdomen demonstrated extensive new metastatic disease, including large masses in the pancreatic region and throughout the abdomen, mediastinal lymphadenopathy, and recurrent right inguinal lymph node enlargement (Fig. 5). An endoscopic biliary stent was placed on September 23, 2024, to relieve obstructive jaundice.

**Table 1:** Timeline of the patient’s clinical course from initial presentation to death.

Time	Event
Jan-24	Initial presentation: painless mass in the right groin
9-Feb-24	Ultrasound-guided core needle biopsy of the right inguinal mass; pathology suggested neuroendocrine carcinoma
23-Feb-24	Whole-body PET/CT scan; no primary lesion identified
23-Mar-24	Surgical excision of the right inguinal mass; pathology confirmed metastatic Merkel cell carcinoma
Sep-24	Onset of abdominal pain, jaundice, and weight loss; imaging revealed widespread metastases; biliary stent placed
8-Oct-24	Peripancreatic biopsy; MCPyV positivity confirmed; diagnosis: Stage IV MCC
21-Oct-24	Initiation of first-line therapy: EP (etoposide + cisplatin) + sintilimab
17-Nov-24	Completion of second cycle; abdominal pain recurred; CT confirmed disease progression
26-Dec-24	Initiation of second-line therapy: FOLFOX + sintilimab
18-Jan-25	Completion of second-line therapy; progressive disease
5-Apr-25	Patient died from progressive disease

CT = computed tomography, FOLFOX = folinic acid + fluorouracil + oxaliplatin, MCC = Merkel cell carcinoma, MCPyV = Merkel cell polyomavirus, PET = positron emission tomography.

**Figure 6.**
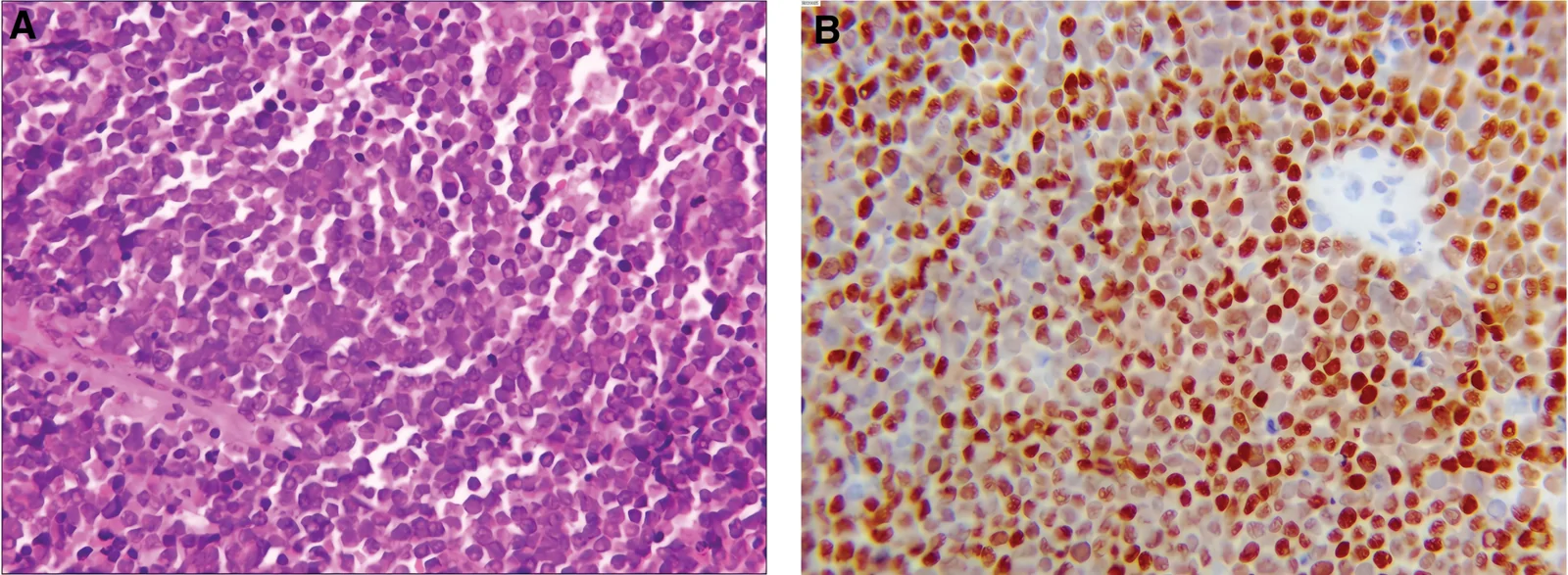
Pathological and immunohistochemical findings of the peripancreatic biopsy. (A) Microscopic examination demonstrated a small round cell malignant tumor, consistent with previous specimens (H&E stain, ×400). (B) Immunohistochemical staining showed positivity for Merkel cell polyomavirus (MCPyV; IHC stain, ×400). H&E = hematoxylin and eosin, IHC = immunohistochemistry, MCPyV = Merkel cell polyomavirus.

On October 8, 2024, an ultrasound-guided biopsy of a peripancreatic mass was performed. Pathology again revealed a small round cell malignant tumor consistent with the previous specimens. To confirm the diagnosis, this biopsy material was sent for additional IHC studies, which demonstrated positivity for MCPyV, special AT-rich sequence-binding protein 2 (SATB2), and INSM1, with retained retinoblastoma protein (Rb) expression (no deletion; Fig. 6). The final diagnosis was Stage IV MCC with metastases to the pancreas, extensive abdominal sites, mediastinum, and right inguinal lymph nodes.

According to the NCCN (National Comprehensive Cancer Network) and CACA (China Anti-Cancer Association) guidelines,^[9,10]^ for metastatic MCC, combination chemotherapy regimens such as etoposide + platinum, EC, or cyclophosphamide + doxorubicin + vincristine may be attempted. Although immune checkpoint inhibitors such as pembrolizumab and avelumab have become standard treatment options in many regions, considering economic factors and drug accessibility, we opted for sintilimab – a programmed cell death-ligand 1(PD-1) inhibitor widely available in China – after thorough communication with patients and obtaining their consent. Systemic treatment was initiated on October 21, 2024, based on the patient’s rapidly progressive visceral disease requiring prompt disease control, with the etoposide + platinum regimen (etoposide 100 mg/m^2^ D1-3, cisplatin 25 mg/m^2^ D1-3) combined with the PD-1 inhibitor sintilimab (200 mg, D1), repeated every 21 days. The patient’s abdominal pain resolved completely after the first cycle. However, following the second cycle (administered on November 17, 2024), abdominal pain recurred. Restaging CT scans confirmed rapid disease progression. Second-line folinic acid + fluorouracil + oxaliplatin was selected given the lack of standard salvage options and the potential activity of platinum-based rechallenge in neuroendocrine tumors. Second-line therapy with the folinic acid + fluorouracil + oxaliplatin regimen (oxaliplatin 85 mg/m^2^ D1, leucovorin 400 mg/m^2^ D1, fluorouracil 400 mg/m^2^ bolus then 2400 mg/m^2^ continuous infusion over 46 hours) combined with sintilimab was administered for 2 cycles (December 26, 2024, and January 18, 2025). This treatment was also ineffective, with progressive worsening of abdominal pain and clinical status. Active antitumor therapy was subsequently discontinued. The patient died from progressive disease on April 5, 2025.

## 3. Discussion

This report describes a 44-year-old male diagnosed with MCC. The diagnosis was based on surgical resection and pathological examination of a right inguinal mass, which presented as the initial symptom. The disease progressed rapidly. Within 6 months after surgery, distant metastases were detected in the pancreas, abdomen, mediastinum, and right inguinal lymph nodes. Notable features of this case include the patient’s relatively young age compared to the typical elderly population affected by MCC, the absence of a primary cutaneous lesion (a clinically uncommon presentation), and the tumor’s aggressive behavior characterized by poor response to both chemotherapy and immunotherapy.

MCC is a rare but highly aggressive neuroendocrine carcinoma that primarily originates from the skin (approximately 10% of patients have no identifiable primary lesion),^[11]^ most frequently occurring in elderly and immunocompromised individuals.^[2]^ While aspects of its pathogenesis remain unclear, evidence indicates a close association with MCPyV.^[3]^ Ultraviolet radiation exposure is also considered a significant etiological factor, particularly in MCPyV-negative cases.^[12,13]^ Diagnosis of MCC is relatively difficult. It requires differentiation from SCLC and other metastatic neuroendocrine tumors. Initially, the case was diagnosed as metastatic small cell carcinoma on biopsy. MCC is characterized by perinuclear dot-like positivity for CK20, while TTF-1 is consistently negative. In this case, CK20 was strongly positive and TTF-1 negative, which rules out the vast majority of small cell lung carcinoma (SCLC). MCPyV immunohistochemistry (anti-MCPyV large T-antigen antibody clone) is highly specific for MCC (positive in approximately 80% of cases), while SCLC and all other neuroendocrine tumors are negative. The positive MCPyV in this case directly confirmed the diagnosis of MCC and indicates the virus-associated subtype (which accounts for the majority). SATB2 is recognized as a specific marker for colorectal carcinoma and bone-derived tumors, with a relatively low expression rate in MCC. However, SATB2 alone cannot diagnose a particular tumor and must be combined with other immunohistochemical markers for auxiliary diagnosis. In the present case, immunohistochemical staining (CK20+, TTF-1−, SATB2+, MCPyV+) suggested metastatic MCC (Table 2). Rb loss on immunohistochemistry itself has no differential significance. Although the majority of MCPyV-positive MCC cases exhibit Rb gene loss, this case retained Rb expression – a known exception observed in approximately 10% to 15% of cases – thus MCC could not be excluded, often due to truncation of the large T antigen (LT) that fails to degrade Rb despite viral positivity. Therefore, Rb retention does not exclude MCC diagnosis.^[11]^ This discrepancy reveals a core pitfall in the differential diagnosis of morphologically similar high-grade neuroendocrine tumors. It also underscores the importance of a combination of immunomarkers. Ultimately, the diagnosis of MCC was confirmed through comprehensive immunohistochemical evaluation. The disease in this patient progressed from onset to death within 14 months, without identifiable skin lesions.^[7]^ This underscores that, in cases of unexplained lymphadenopathy, MCC should be considered after excluding other common malignancies, warranting prompt pathological diagnosis and comprehensive evaluation to guide timely and individualized management.

**Table 2:** Comparison of typical immunophenotypic features among MCC, small cell lung carcinoma, and other metastatic neuroendocrine tumors.

Marker	Merkel cell carcinoma (MCC)	Small cell lung carcinoma (SCLC)	Other metastatic neuroendocrine tumors (e.g., LCNEC, carcinoid)
CK20	Positive (perinuclear dot-like, >80%)	Negative (<5% may show focal weak positivity)	Negative (rare exceptions, e.g., some gastroenteropancreatic NETs may be positive)
TTF-1	Negative (nearly 100%)	Positive (approximately 80–90%, strong nuclear)	Partially positive (LCNEC ~30–50%; carcinoid often negative)
SATB2	Positive (approximately 60–80%, diffuse or focal)	Negative (very rare weak positivity)	Negative (but colorectal NETs may be positive; correlation with clinical context needed)
MCPyV	Positive (approximately 80%, nuclear or cytoplasmic, clone CM2B4)	Negative	Negative
Rb protein	Lost (in MCPyV + cases) or retained (in MCPyV − cases)	Lost (>90% due to RB1 mutation inactivation)	Variable: LCNEC often lost (~60%), carcinoid usually retained
Syn/CgA	Both positive (diffuse strong)	Positive (usually Syn stronger than CgA)	Positive (carcinoid: both positive; LCNEC: Syn predominant)
CD56	Positive	Positive (strong and diffuse)	Positive (may be weak or negative in carcinoid)
CK7	Negative or focal weak positivity	Positive (approximately 40–60%, patchy)	Carcinoid often positive; LCNEC variable
Ki-67	>20% (often >50%)	>50% (often >80%)	LCNEC > 20%; carcinoid < 20%

The above data are compiled from clinical practice. Actual positivity rates may vary depending on reagents and interpretation thresholds. Loss of Rb expression refers to complete absence by immunohistochemistry or only scattered weak positivity.

CD56 = cluster of differentiation 56, CgA = chromogranin A, CK7 = cytokeratin 7, CK20 = cytokeratin 20, CM2B4 = anti-MCPyV large T-antigen antibody clone, Ki-67 = marker of proliferation Ki-67, LCNEC = large cell neuroendocrine carcinoma, MCC = Merkel cell carcinoma, MCPyV = Merkel cell polyomavirus, NET = neuroendocrine tumor, Rb = retinoblastoma protein, SATB2 = special AT-rich sequence-binding protein 2, SCLC = small cell lung carcinoma, TTF-1 = thyroid transcription factor 1.

Note: the above data are compiled from clinical practice. Actual positivity rates may vary depending on reagents and interpretation thresholds. Loss of Rb expression refers to complete absence by immunohistochemistry or only scattered weak positivity.

For localized MCC, surgical resection remains the primary treatment.^[14,15]^ However, due to its aggressive nature, high recurrence rate, and tendency for early metastasis,^[5]^ surgery alone is often insufficient. Radiotherapy is commonly combined with surgery to reduce local recurrence or serves as a primary modality for inoperable patients.^[16]^ Although MCC exhibits initial sensitivity to chemotherapy, responses are typically transient, and resistance develops rapidly.^[17]^ Consequently, chemotherapy is primarily utilized for palliative management of advanced or metastatic disease.^[18]^

Cellular immunity plays a pivotal role in MCC pathogenesis and treatment.^[19,20]^ While MCC can elicit a robust immune response, it frequently employs mechanisms to evade immune surveillance, a rationale supporting immunotherapy.^[21]^ In recent years, immune checkpoint inhibitors have demonstrated significant efficacy in treating MCC.^[22,23]^ Notably, the anti-PD-1 antibody Pembrolizumab and the anti-PD-L1 antibody Avelumab have received FDA approval for metastatic MCC.^[24,25]^ However, in the present case, the patient achieved only a very brief response (approximately 1 month) to a regimen combining sintilimab (a PD-1 inhibitor) with chemotherapy. This was followed by rapid disease progression, widespread dissemination of metastases, resistance to systemic therapy, and death approximately 14 months after presentation.

Several factors may explain the poor outcome in this case. Adjuvant therapy was not administered after the initial surgical resection due to the absence of visible residual disease and the lack of validated adjuvant treatment protocols for MCC at the time of surgery. Additionally, the patient declined further intervention given the absence of symptoms. Systemic treatment was only initiated after extensive tumor dissemination had already occurred, which likely limited its efficacy. This course reflects the heterogeneous and sometimes refractory nature of advanced MCC.

Several studies have reported that patients with nodal MCC without an identifiable primary lesion may experience better survival outcomes than stage-matched patients with known primary tumors.^[8,26-28]^ This observation has been attributed to spontaneous immune-mediated regression of the primary lesion and enhanced host antitumor immunity.^[8,29,30]^ The present case stands in stark contrast to this pattern. A plausible explanation for this discrepancy lies in the heterogeneity of MCC. It may also be due to immune evasion mechanisms, such as β-catenin pathway activation or MHC-I downregulation. Intrinsic molecular subtypes (e.g., with TP53 mutations) may override the usual immunologic advantage.^[31]^ Research into the mechanisms underlying primary and acquired resistance to immunotherapy,^[32]^ as well as the development of novel agents such as MCPyV-targeted therapies,^[33]^ is ongoing and represents a crucial avenue for improving outcomes.

In conclusion, this case exemplifies a rare and challenging presentation of occult-primary MCC, along with young age and highly aggressive, treatment-resistant behavior. It reinforces the necessity of a high index of suspicion and meticulous pathological workup for accurate diagnosis. Furthermore, it underscores that despite significant therapeutic advances with immunotherapy, a subgroup of patients with advanced MCC continues to have a poor prognosis due to rapidly progressive disease. Continued research into the tumor’s biology and the development of more effective treatment strategies are imperative for this patient population.

## Patient perspective

A patient perspective could not be obtained, as the patient died from progressive disease prior to manuscript preparation. Written informed consent for publication was obtained from the patient’s family members.

## Acknowledgments

Acknowledgments are extended to the Department of Pathology at the General Hospital of Ningxia Medical University and China-Japan Friendship Hospital for providing the pathological data.

## Author contributions

**Data curation:** Wen Xiao, Lijun Su.

**Writing – original draft:** Na Luo.

**Writing – review & editing:** Ping Chen.
